# Washing the Face and Hands

**Published:** 1869-02

**Authors:** 


### WASHING THE FACE AND HANDS

Is performed by millions every day, and yet perhaps not a dozen in a million will do it right. The common practice is to take a basin of cold water, catch up a double handful of water, dash it into the face and rub it vigorously, thus rubbing all the matters of the soiled hands which have accumulated during the night into the skin of the face.

It is a great luxury to wash the hands thoroughly and well with soap and warm water among the very last things on going to bed at night. The cleanliest person will often find that a tea-cupful of warm water will be soiled by the operation, and the same will again occur the first thing in the morning, since in addition to adventitious causes there is during sleep an exudation of an oily substance through the pores of the skin, and to this floating dust of the rooms, furniture and clothing will adhere.

It is one of the inestimable blessings of city life that both warm and cold water are at hand at all hours, day and night.

Take a tea-cupful of warm water, not more, and with soap make a lather, with which wash the hands thoroughly, not forgetting under the ends of the finger-nails, and dabble in the water until every particle of accumulation is removed. This is better than to use a brush, because the hard bristles will irritate and harden the tender skin under the ends of the finger-nails. Scraping out the dust with a pen-knife is an inexcusable violence, and an indecency, too, when done in company. Next rince the hands in an abundance of water until all the soap is thoroughly removed. The face may then be washed in another supply of water, warm or cool, according to the taste of the individual. Warm water is better, as it dissolves more readily any accumulations about the eyes or ears, and then with an instantaneous rinsing of the face in cold water, the work is done; and then your hands and face are clean enough to pat the face and kiss the cheek of the one you love best as a morning salutation.

